# Imatinib-Induced Pleuro-Pericardial Effusion and Atrial Fibrillation: An Unusual Side Effect Following the Treatment of a Rare Gastrointestinal Tumor

**DOI:** 10.7759/cureus.37727

**Published:** 2023-04-17

**Authors:** Neel N Patel, Sharan Jhaveri, Gashaw Hassen, Chaithanya Avanthika, Sajid Siddiq

**Affiliations:** 1 Internal Medicine, New York Medical College/Landmark Medical Center, Woonsocket, USA; 2 Internal Medicine, Smt. NHL Municipal Medical College, Ahmedabad, IND; 3 Internal Medicine, Mercy Medical Center, Baltimore, USA; 4 Pediatrics, Icahn School of Medicine at Mount Sinai, Queens Hospital Center, New York, USA; 5 Medicine and Surgery, Karnataka Institute of Medical Sciences, Hubli, IND; 6 Cardiology, New York Medical College/Landmark Medical Center, Woonsocket, USA

**Keywords:** rare cause of pleural effusion, recurrent pericardial effusion, atrial fib, gastrointestinal stromal tumor (gist), imatinib

## Abstract

Gastrointestinal stromal tumors (GISTs) are one of the most prevalent non-epithelial tumors of the GI mesenchyme. While stromal tumors account for less than 1% of all malignancies, a knowledge of their etiology and signaling pathways can aid in identifying new molecular targets for the potential development of therapeutics. One of the drugs that have shown remarkable action against GIST is imatinib, a tyrosine kinase inhibitor (TKI).

We present a case of a female patient with a long-term history of heart failure (HF) with preserved ejection fraction (EF) and minimal pericardial effusion who had recently started imatinib therapy and was hospitalized after new-onset atrial fibrillation (AF) and the development of significantly increased pericardial and pleural effusion. She had been diagnosed with GIST a year ago and started on imatinib. She presented to the ER with complaints of left-sided chest pain. ECG revealed a new AF. The patient was started on rate control and anticoagulation. After a few days, she returned to the ER with complaints of shortness of breath (SOB). The patient was found to have pericardial and pleural effusions on imaging. Fluids from both effusions were aspirated and sent to pathology to rule out malignancy. The patient developed recurrent bilateral pleural effusions after discharge, which were later drained on subsequent hospitalization.

Although imatinib is generally well tolerated, it does cause both AF and pleural/pericardial effusions in rare cases. In such cases, it is essential to perform a thorough workup to rule out other possibilities such as metastasis, malignancy, or infection.

## Introduction

Gastrointestinal stromal tumors (GISTs) are rare mesenchymal neoplasms of the GI tract; they are most commonly seen in the stomach (60-70%) and predominately affect older adults (median age at diagnosis: 64 years) with an annual incidence of seven to eight cases per million population in the United States [1]. Imatinib, a tyrosine kinase inhibitor (TKI), which was initially approved as a prototype drug for chronic myeloid leukemia (CML), has been recognized as a revolutionary drug of choice in the effective treatment of GISTs [2]. Imatinib is a generally well-tolerated chemotherapeutic drug having a low incidence of severe side effects [3]. However, there have been a few case reports on the rare side effects of imatinib, such as pericardial effusion in a child and pleural/pericardial effusion in an adolescent [4]. In fact, there is also a published case report of pleural effusion in an 88-year-old male who underwent GIST treatment with imatinib [5]. We report a rare case of pleural and pericardial effusion in an 83-year-old female diagnosed with GIST at the cardia of the stomach and treated with imatinib. Clinicians should be aware of this side effect as it could be mistaken for other similar presentations such as metastasis or infection.

## Case presentation

An 83-year-old female patient with a long history of minimal pericardial effusion for at least the last two years was hospitalized two weeks ago after an echocardiogram revealed a recent development of significantly increased pericardial effusion associated with shortness of breath (SOB).

Her past medical history was remarkable for a 65 x 37 mm, oval, and intramural (subepithelial) GIST, which had been diagnosed a year ago after she presented with progressively worsening dysphagia, for which she was started on 200 mg/day imatinib immediately by an outpatient GI oncologist. Her imatinib dose had been later increased to 400 mg/day after she tolerated her first week of treatment. She had refused surgical intervention after four months of imatinib treatment when her tumor showed a stable pattern and was found eligible for resection. But, she had continued her imatinib course for another six months until three weeks ago when she complained about mid-sternal chest pain associated with palpitations. She was diagnosed with new-onset atrial fibrillation (AF), type 2 non-ST-elevation myocardial infarction (NSTEMI), and congestive heart failure (CHF) with an ejection fraction (EF) of 55%. Her chest X-ray during initial admission showed cardiomegaly, pulmonary congestion, probable underlying pulmonary edema, focal opacities in the left lower lobe, and bilateral small pleural effusions more significant on the left side. The patient was started on metoprolol 25 mg every eight hours for rate control, and furosemide 40 mg oral daily for diuresis. She was put on heparin initially for anticoagulation, which was later transitioned to apixaban after the echocardiogram was unremarkable for any new wall motion abnormalities at EF of 55%. She was then discharged home after the chest pain resolved and her EF normalized with continued apixaban, metoprolol, and furosemide reduced to 20 mg/day oral.

**Figure 1:**
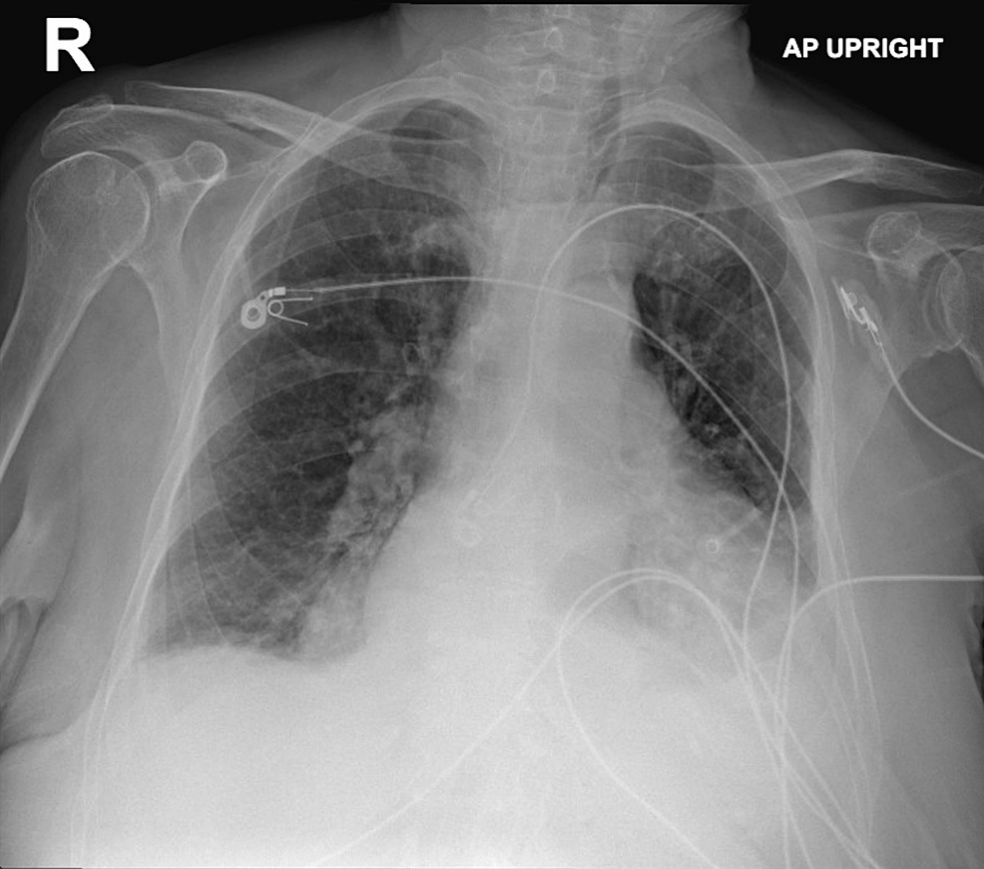
Initial chest X-ray showing cardiomegaly and pleural effusion

**Figure 2:**
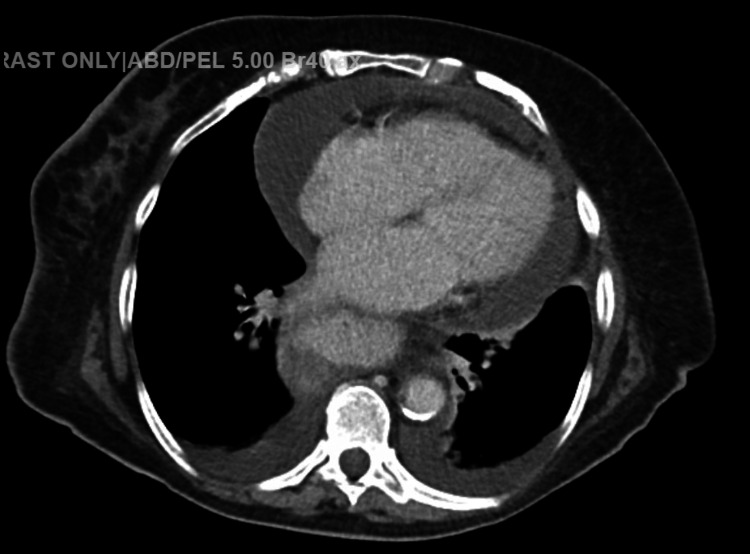
Initial CT angiogram, suggestive of pleural and pericardial effusion CT: computed tomography

A week later, the patient returned to ED with a complaint of diarrhea and left-sided abdominal pain. Also, she had SOB during which further workup revealed controlled AF, significant cardiomegaly compared to prior X-ray, and a new moderate to large pericardial effusion based on a CT angiogram; she was admitted for further workup and inpatient management. Chest X-ray and the CT angiogram suggestive of pleural and pericardial effusions are shown in Figures 1-2

**Figure 3:**
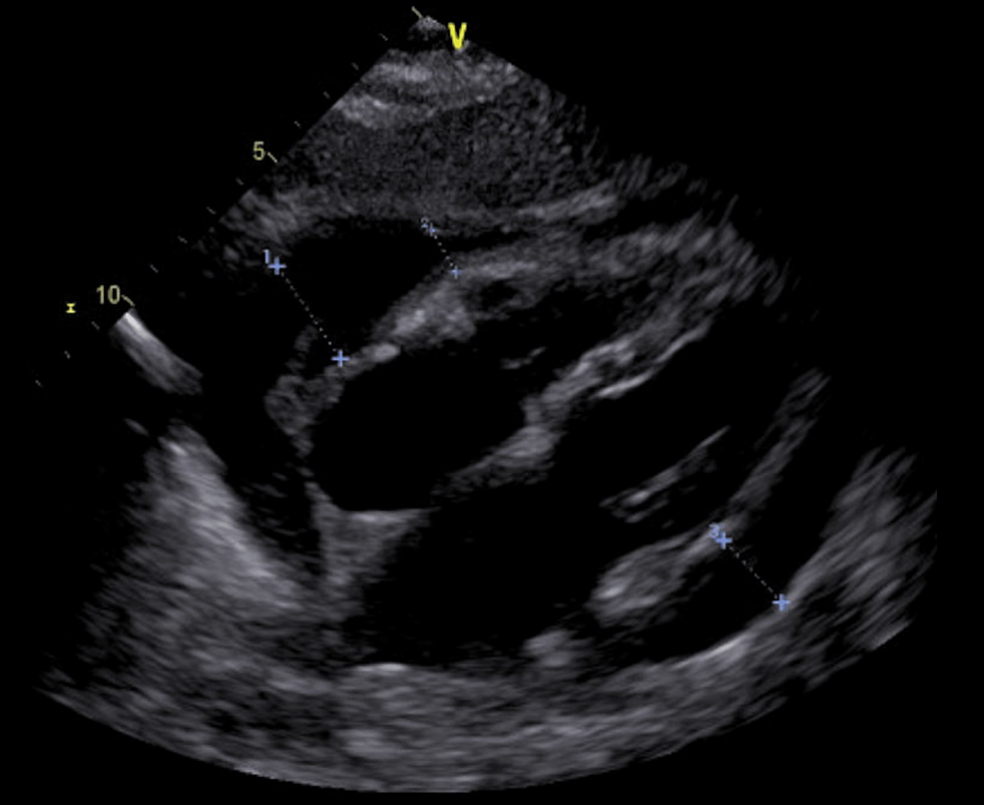
Echocardiogram showing moderate to severe collection of pericardial fluid

A repeat echocardiogram (Figure 3) showed moderate-severe circumferential effusion with no significant change compared to the last echo, a respiratory variation on the right ventricular (RV) inflow of 28%, and no evidence of RV collapse.

Pericardiocentesis was performed after a repeat of limited echocardiogram confirmed moderate to large pericardial effusion revealing 500 ml of bloody pericardial fluid. Right heart catheterization findings were as follows: RA: 22, RV: 42/20, PA: 42/30, PCWP: 30-35, and filling pressures were elevated in addition to diastolic equalization. A pericardial drain was placed. The next day, a repeat limited echo (s/p pericardiocentesis) showed minimal circumferential pericardial effusion and fibrinous thickening of the pericardium. The respiratory variation in RV inflow was approximately 30%. The pericardial drain had additional 100 ml output overnight before it was removed.

After pericardiocentesis, the patient continued to have SOB, and CXR findings were consistent with moderate left pleural effusion associated with atelectasis and a slight worsening of pleural effusion as compared to the previous one. A further ultrasound-guided left thoracentesis by an interventional radiologist drained 1010 ml of yellow/straw color fluid, which resulted in a significant improvement of SOB and her symptoms improved dramatically.

Further cytological studies of both the pleural and pericardial fluid were done. The smear of the pericardial fluid showed a majority of RBC and very rare atypical cells with histiocytes. Cytologic analysis of the pleural fluid revealed atypical cells along with histiocytes, mesothelial cells, mixed inflammatory cells, and abundant red blood cells. Block preparation and ancillary studies were recommended to rule out malignant cells given the history of melanoma, breast carcinoma, and malignant GIST.

## Discussion

The diagnosis of a GIST before confirmatory biopsy can be complicated, requiring significant contributions from radiologists as well as a high index of suspicion since nearly 20% of patients present with non-specific GI symptoms [6]. Treatment options depend on various factors and range from surgical resection to various novel antineoplastics. GISTs used to have a poor prognosis and were known to be notoriously resistant to chemotherapy, but the introduction of TKIs has brought about a radical shift in disease outcomes.

TKs play a crucial role in multiple stages of the cell cycle and are important for signal transduction across the body. Although TKs are indispensable for normal function, certain genetic aberrations of these TKs make for great molecular targets for treating cancers. For instance, the BCR-ABL TK is seen in CML. The primary target of imatinib is the ATP binding site of TK. By inhibiting this enzyme, it prevents the phosphorylation of proteins involved in the BCR-ABL signal transduction cascade [7]. Apart from BCR-ABL, imatinib also acts on other molecular targets such as platelet-derived growth factor (PDGF) alpha/beta as well as KIT. The latter is a growth factor with TK activity. Gain of function mutations in c-KIT, the proto-oncogene that encodes KIT, are commonly seen in a large proportion of GISTs and contribute to their development [8]. These mutations cause ligand-independent TK activity, leading to uncontrolled cell proliferation and stimulation of downstream signaling pathways. The first recorded attempt to treat metastatic GIST with imatinib (or STI571 as it was known back then) by Joensuu et al. was published in the New England Journal of Medicine (NEJM) in 2001 [8]. This was followed by multiple clinical trials to test the efficacy and safety profile of imatinib. Today, imatinib is the standard first-line therapy for metastatic GIST, due to its effectiveness and relatively limited contraindications [9].

As imatinib targets multiple kinases, its side effect profile is quite diverse, the most obvious one being the impact on immune cells and immunity. Neutropenia, inhibition of CD34 dendritic cells, and reduced T cell and monocyte proliferation are some of the commonly cited adverse effects of imatinib on the immune system [10]. Skin rashes, dyspepsia, fatigue, etc are other commonly encountered adverse drug effects. The one most relevant to our patient is severe fluid retention (FR).

As cataloged by Kim et al., FR associated with imatinib is broadly divided into two types (Table 2) [11].

**Table 1:** Fluid retention (FR) associated with imatinib

	Acute progressive FR	Intermittent/steady FR
Characteristics	Acute aggravation with rapid improvement	Occasional or mildly persistent FR
Median time to max FR from drug initiation/escalation	1.9 months	Any time during stable dosing, or mild FR after escalation

Subcutaneous edema, especially in the periorbital regions and the lower extremities, seems to be the most consistent presentation of FR. The mechanism of this FR remains unclear, with several hypotheses providing different rationales [12].

Of particular interest to our case are the manifestations of pleural and pericardial effusion. The frequency of severe FR associated with imatinib is relatively lower when compared to newer second- and third-generation TKIs, especially dasatinib [13]. Regardless, several presentations have been documented. Imatinib-induced pleural effusion happens to be dose-dependent, with a higher incidence seen in patients treated with more than 400 mg a day, as seen with our patient [14]. Apart from symptomatic treatment, usually provided by diuretics or tapping, determining the cause of the effusion is equally important for further management.

The incidence of cardiovascular adverse effects is seen to be far higher in second- and third-generation TKIs when compared to imatinib [15]. It is interesting to note that pericardial effusions in imatinib-treated patients rarely occur on their own, and are associated with pleural effusion [16]. Moreover, these patients may have malignant effusions, but imatinib cessation resolves these complications, suggesting that imatinib therapy precipitates effusions in some cases [13].

It is also worth discussing AF, which complicated the clinical presentation of this case. AF is a cardiovascular toxicity side effect in 0.55% of patients using imatinib [17]. Imatinib-induced AF is also reported in some cases of patients receiving TKIs as chemotherapy [18]. The prevalence of AF shows an increasing trend with age. A European population-based prospective cohort study on the prevalence of AF showed an overall prevalence of 5.5%, which increased from 0.7% in patients aged 55-59 years to 17.8% in those aged 85 years and above [19]. The presence of chronic heart disease is also closely associated with a higher incidence of AF as shown in multiple cases of coexisting AF and heart failure (HF) in which AF both precedes and follows HF with both preserved and reduced EF. Large pericardial effusion can also cause AF [20].

## Conclusions

Imatinib, despite being the first-line therapy for metastatic GIST, has a diverse side effect profile. FR is a known adverse effect of TKIs, which may cause third-space fluid accumulation, Imatinib-induced pleural effusion, and pericardial effusions are rare but potentially serious adverse effects. The side effect profile of patients on TKIs must be closely monitored. A new pleural effusion should not be assumed to be due to a worsening tumor or infection, and imatinib should be kept in mind as a less common yet important differential.
